# Supplementary figures and images for: Stabilization of PIM Kinases in Hypoxia Is Mediated by the Deubiquitinase USP28
Source: Cells. 2022 Mar 16;11(6):1006. doi: 10.3390/cells11061006 (PMC8947361; doi:10.3390/cells11061006)

Article

# Stabilization of PIM Kinases in Hypoxia Is Mediated by the Deubiquitinase USP28

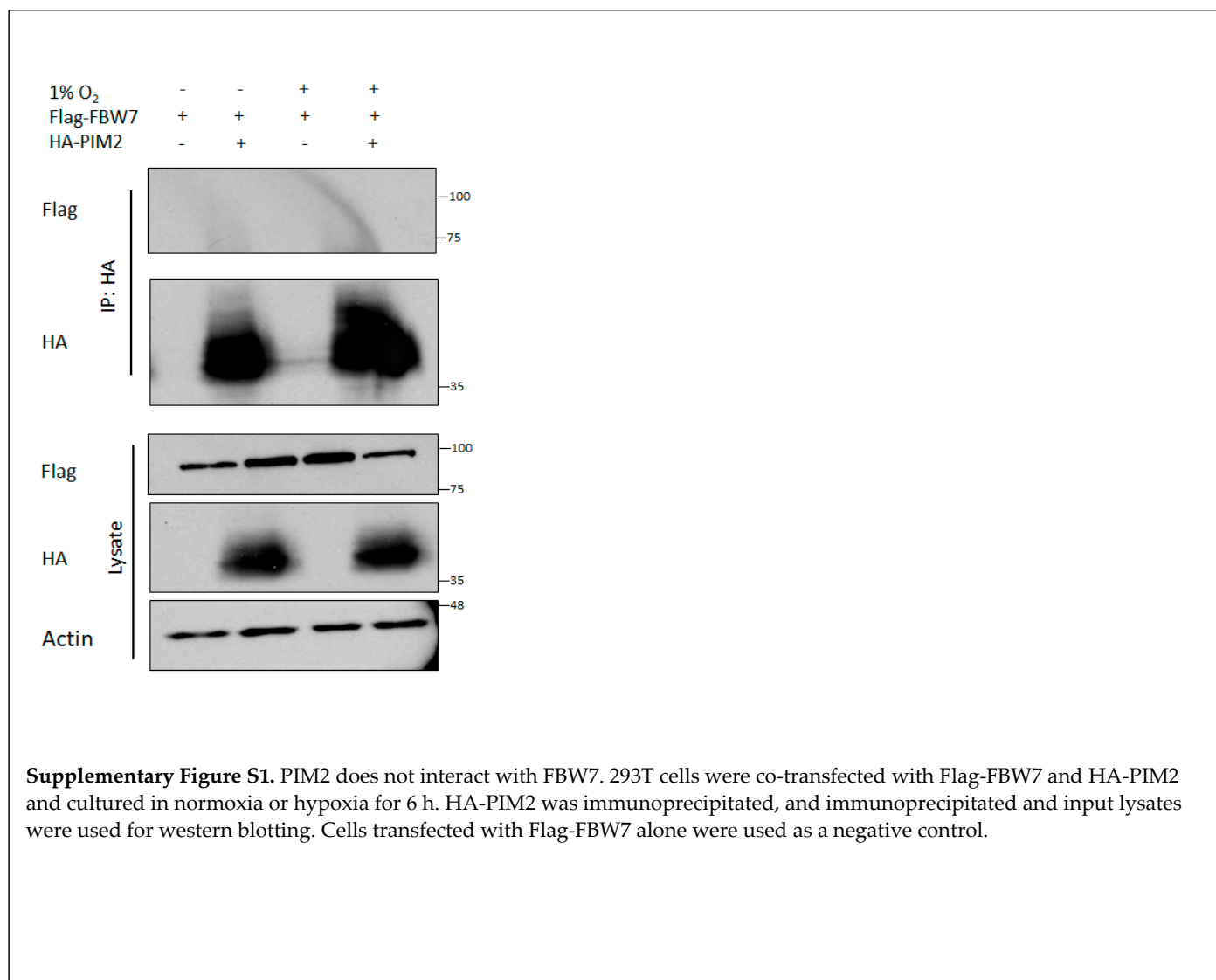

Supplement: Supplementary file 1 [file cells-11-01006-s001.zip › cells-1587810-supplementary.pdf]
